# An Overview of Signal Processing Techniques for Remote Health Monitoring Using Impulse Radio UWB Transceiver

**DOI:** 10.3390/s20092479

**Published:** 2020-04-27

**Authors:** Faheem Khan, Asim Ghaffar, Naeem Khan, Sung Ho Cho

**Affiliations:** 1 Department of Electronics and Computer Engineering, Hanyang University, 222 Wangsimni-ro, Seongdong-gu, Seoul 04763, Korea; faheemkhan@hanyang.ac.kr (F.K.); asimghaffar@hanyang.ac.kr (A.G.); 2 Department of Electrical Engineering, Engineering University, Peshawar 25000, Pakistan; nkhan@uetpeshawar.edu.pk

**Keywords:** vital signs, IR-UWB radar, algorithm, respiration rate, heart rate, motion detection, sleep monitoring, fall detection

## Abstract

Non-invasive remote health monitoring plays a vital role in epidemiological situations such as SARS outbreak (2003), MERS (2015) and the recently ongoing outbreak of COVID-19 because it is extremely risky to get close to the patient due to the spread of contagious infections. Non-invasive monitoring is also extremely necessary in situations where it is difficult to use complicated wired connections, such as ECG monitoring for infants, burn victims or during rescue missions when people are buried during building collapses/earthquakes. Due to the unique characteristics such as higher penetration capabilities, extremely precise ranging, low power requirement, low cost, simple hardware and robustness to multipath interferences, Impulse Radio Ultra Wideband (IR-UWB) technology is appropriate for non-invasive medical applications. IR-UWB sensors detect the macro as well as micro movement inside the human body due to its fine range resolution. The two vital signs, i.e., respiration rate and heart rate, can be measured by IR-UWB radar by measuring the change in the magnitude of signal due to displacement caused by human lungs, heart during respiration and heart beating. This paper reviews recent advances in IR- UWB radar sensor design for healthcare, such as vital signs measurements of a stationary human, vitals of a non-stationary human, vital signs of people in a vehicle, through the wall vitals measurement, neonate’s health monitoring, fall detection, sleep monitoring and medical imaging. Although we have covered many topics related to health monitoring using IR-UWB, this paper is mainly focused on signal processing techniques for measurement of vital signs, i.e., respiration and heart rate monitoring.

## 1. Introduction

In the year 2002, the Ultra-Wide Band (UWB) regulations permitted the unlicensed operation in the frequency range of 3.1 to 10.6 GHz. After unlicensed operation, UWB technology was used for wireless communication and radar applications [1]. Impulse Radio Ultra Wideband (IR-UWB) wireless systems are generally based on the transmission and reception of sub-nanosecond pulses without carriers, or modulated short pulses with carriers [2]. Since it transmits the pulses with very low power [3], it is completely health-risk free and, hence, it can be used on daily basis. Recently, IR-UWB technology has been used in many applications due to its characteristics such as robustness in a harsh environment, accurate ranging at the level centimeters, less power consumption and good object penetration capacity [4]. Impulse radar has been used in many fields such as localization [5], medical [6,7,8,9,10,11,12,13,14,15,16,17,18,19,20,21,22,23,24], multihuman detection [25,26], gesture recognition [27,28,29,30], imaging [31,32], tumor detection [33,34,35,36,37] and people counting [38]. Radar waveform optimization has been studied by many researchers due to its importance in radar hardware design. Constant-modulus waveform design was considered in Reference [39] for extended target detection. 

Recently, radar sensors have been used in a network fashion to improve estimation and positioning. A study performed by Leem et al. used three radar sensors for digit writing in mid-air [29]. An authentication method was introduced using four radar sensors and the Convolutional Neural Network (CNN) [40]. A network of radio sensors may be used for accurate indoor localization and tracking of objects as discussed in detail in Reference [41]. 

Due to its low cost, high range resolution and low power requirements, radar sensors are not only used for indoor sensing [4] and vehicular applications, but they have also been used in smart phones. Researchers in Reference [42] have developed a miniature low-cost radar for smartphone applications. The small size radar may be used for object detection, tracking and imaging. A mobile phone giant, Google, has launched a Soli based gesture recognition application in its new smartphone Google Pixel 4. In the future, more and more radar-based applications, such as detection, activity recognition and vital signs monitoring, will be embedded in hand phones manufactured by smartphone giants. 

Due to the property of electromagnetic backscattering [43], radar can wirelessly detect both chest-wall movements caused by respiration and weak heart beats. The UWB motion sensor was developed by McEwan at the Lawrence Livermore National Laboratory (LLNL) [44]. An IR-UWB radar-based vitals monitoring system may be used at home as well as for continuous vitals monitoring of people during routine activities, i.e., during working on computer, vehicle driving or resting/sleeping in home. The vital signs of a driver are very important as it can be helpful for drowsiness detection and, hence, it can prevent car accidents [30]. Fall detection is also very hot research topic these days, and radar can detect falls without invasion of privacy unlike camera, so it can be helpful for monitoring elderly or sick people at their homes. Moreover, vital signs measurement of neonates and burned people can be facilitated by radar due to its non-invasive nature. 

Recently, many researchers have shown interest in the non-invasive vital signs monitoring of humans due to the fact that it is easy to monitor a person wirelessly than using wired conventional electrocardiography (ECG), pulse oximetry and innovative wearable devices. These wired devices have disadvantages such as limiting mobility of patients and spread of contagious infections between patients and hospital staff members [45]. The non-invasive monitoring is much more useful than complicated wired connections such as monitoring through ECG because, in some situations, it is hard to use wires, such as for an infant or patients who got burned in some accident or people buried during some building collisions [4]. Moreover, the bed capacity become a problem as more elderly people get hospitalized due to various physical problems [46]. For many developed countries such as Japan, the aging population and costs of healthcare become leading concerns [47]. The government is trying to reduce the hospital costs, and one possible solution is home healthcare. The deployment of an IR-UWB vital measurement system may enable proactive monitoring of patients in home and, in aging people, it can reduce the healthcare cost by shifting a portion of patients from hospital to homes [48]. Impulse radar is one of the hardware devices used for non-invasive vital signs monitoring because of the higher range resolution and better penetration capabilities. The radar is mainly used to monitor the motion of the chest of a human, and the repeated motion of chest due to respiration and heart beating is captured by the radar signal reflected from a body. At rest, a human heart can displace the chest by 0.08 mm [49], and the respiration can cause displacement up to several millimeters depending on the person [50]. The periodic displacement changes of the chest due to heart and lungs is measured from the reflected radar signals.

In this paper, we have discussed the hardware setup and methodology of vital signs monitoring by the state of art techniques. Then we have presented the contributions of different authors regarding the vital signs of a stationary human target. The literature about vital signs of non-stationary humans are also elaborated in detail. Next, we have shown the vital signs for particular applications by different researchers, such as for home, hospitals and cars. Then different feasibility and case studies regarding vital signs are presented. The main focus of this research is on vital signs monitoring using IR-UWB radar; therefore, most sections of the paper (Section 2, Section 3, Section 4, Section 5, Section 6, Section 7, Section 8 and Section 9) are related to vital signs. In Section 10, we have presented briefly the medical imaging and fall detection applications of an IR-UWB radar sensor in order to have a broader perspective of the technology. A related paper [51] has discussed radar-based health monitoring, but the main focus is on hardware optimization research, and the signal processing techniques are not covered in detail. The main contribution of this work is that it reviews most of the research regarding the signal processing techniques for health monitoring using IR-UWB radar. Secondly, we have explored the step by step signal processing methodology for vital signs measurements, and we have overviewed the literature and classified the research work according to applications. Therefore, this paper provides a tutorial overview of the work done in the vital signs through IR-UWB radar. 

## 2. Mathematical Model

In References [4,52], the vital signals reflected from the human subject are mathematically represented and, by using the Fast Fourier Transform (FFT), it is shown that the reflected signal contains the breathing and heart beat signal components. In addition to Heart Rate (HR) and Respiration Rate (RR), harmonics of respiration and heart rate are present in the signal. To cancel the harmonic components, researchers have used the Moving Target Indicator (MTI) filter algorithm. When the signal is transmitted from the transmitted antenna, a part of the signal is reflected back due to the high reflectivity of the human body [4]. The time of flight of the signal is denoted by $\tau_{0},$ and it depends on the distance $d_{0}$ between the antenna and the human sitting in front of it. The following equation represents the instantaneous distance of the chest wall at time ’t’:

(1) $$d\left(t\right)=d_{0}+m\left(t\right)\;=d_{0}+m_{b}\sin\left(2\times \pi \times f_{b}\times t\right)+m_{h}\sin\left(2\times \pi \times f_{h}\times t\right).$$

In the above equation, $m_{b}$ and $m_{h}$ are the respiration and heartbeat amplitudes. Following the mathematical model as described in [11,47], it can be found that Fourier Transform in slow-time is:

(2) $$Y\left(f,\tau_{0}\right)=A\overset{\infty }{\underset{k=-\infty }{\sum }}\overset{\infty }{\underset{l=-\infty }{\sum }}C_{kl}\delta \left(f-kf_{b}-lf_{h}\right)$$

where

(3) $$C_{kl}=\int_{-\infty }^{\infty }P\left(v\right)J_{k}\left(\beta_{b}v\right)J_{l}\left(\beta_{h}v\right)dv.$$

In the above equations, $\beta_{b}=4\pi m_{b}/c$ and $\beta_{h}=4\pi m_{h}/c$. The spectrum in Equation (3) comprises of discrete delta functions centered at frequencies $f_{b}$ and $f_{h},$ and the intermodulation products of these two frequencies.

## 3. Methodology

### 3.1. Hardware Setup

**Figure 1:**
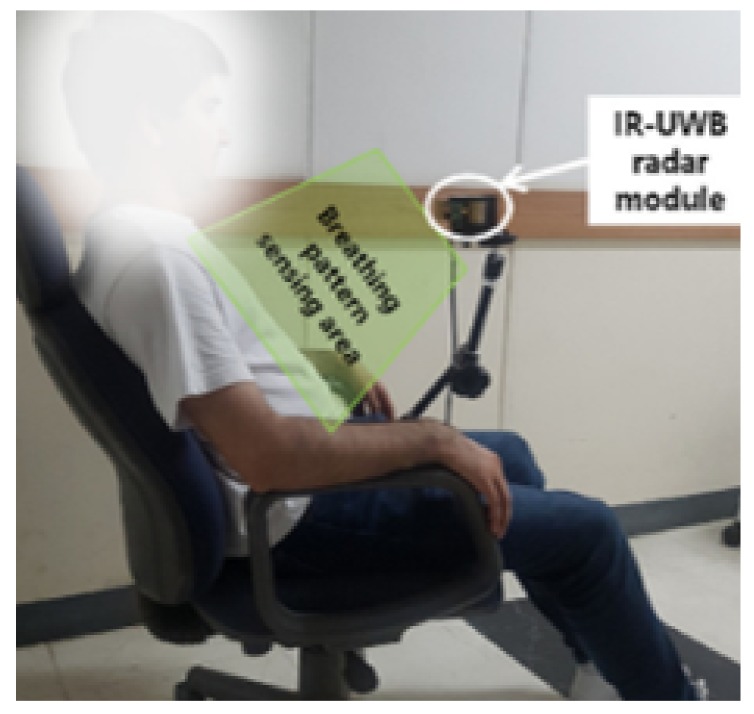
Experimental setup.

The methodology of state-of-the-art vital signs is discussed in this section. The simple hardware setup consists of a radar sensor with transmitter and receiver antenna, which are directed at the human as shown in Figure 1 [4]. The signal reflected from the human chest is received by the radar and then signal processing algorithms are used to remove the noise, motion artefacts and frequency harmonics to find out RR and HR. As the human body is highly reflective, a portion of the transmitted signal is reflected back. The reflected signal contains clutter as well as information related to the motion of the lungs and heart. Clutter refers to the unwanted signal reflected from objects in the environment. The motion part contains the frequency of respiration and heart rate. Normal range of RR and HR are 12–16 and 60–100 cycles per minute, respectively [9]. The raw signal from the human body contains clutter as well as RR and HR information. After clutter removal, waveforms are combined in a matrix form. The size of the matrix depends on the observation interval. Then Fourier/wavelet transform is applied to the matrix to get the spectrum of the signal. As the HR and HR have different and non-overlapping frequency range, it is easy to find the RR from the spectrum. However, the breathing harmonics may appear in the frequency range of HR, and they even have a stronger magnitude than HR; therefore, to get the accurate HR value, researchers have eliminated the breathing harmonics using different techniques such as using the notch filter to remove the multiples of breathing frequencies form the vital signal spectrum [4]. The disadvantage of a notch filter based solution is that it can even suppress HR if the breathing harmonics are located very close to the HR. 

### 3.2. Pre-Processing of the Radar Signals before Vital Signs Extraction

#### 3.2.1. Signal Model

The transmitter in the IR-UWB radar transmits narrow band pulses and the signal reflected from the target object is received by the receiver antenna. The static UWB channel model that was proposed by [53] is given in Equation (4):

(4) $$h\left(\tau \right)=\overset{L}{\underset{j=1}{\sum }}a_{j}p_{j}\left(\tau -n_{j}\right)\;\;$$

(5) $$h\left(t,\tau \right)=\overset{L}{\underset{j=1}{\sum }}a_{j}\left(t\right)p_{j}\left(\tau -n_{j}\left(t\right)\right)\;.\;\;$$

In Equations (4)–(5), ’τ’ represents time delay, and ‘*t*’ is time elapsed. Channel model $h\left(t,\tau \right)$ is the superposition of the ‘*L*’ strongest scattering paths. Path ‘*j*’ is specified by the time of arrival of that path $n_{j}\left(t\right),$ the path amplitude $a_{j}$ and the path waveform $p_{j}\left(\tau -n_{j}\right)$. 

#### 3.2.2. Clutter Removal

The reflected signal contains both the vital signal and the clutter from the environment, as well as the static parts of the human body. The signal part due to unwanted clutter should be removed from the reflected signal. A simple technique based on loopback filter is used to remove clutter signal, which is defined by the following Equation (6) [54].

(6) $$C\left(t\right)=\alpha \cdot C\left(t-1\right)+\left(1-\alpha \right)\cdot x\left(t\right)\;\;=C\left(t-1\right)+\left(1-\alpha \right)\cdot \left(x\left(t\right)-C\left(t-1\right)\right)\;=C\left(t-1\right)+\left(1-\propto \right)\;.\;s^{\prime }\left(t\right)\;$$

**Figure 2:**
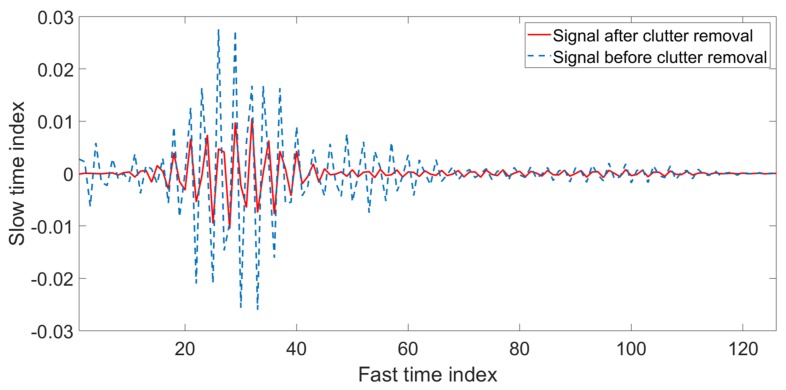
Received signal before and after clutter removal.

In the above equations, the symbol ∝ represents a constant value that is used for weighing, while $C\left(t\right)$ is clutter signal and $s’\left(t\right)$ is the background subtracted signal. Figure 2 shows an example of a signal that contains clutter and, after applying the background subtraction filter, the clutter is removed from the signal. 

In literature, loop back filter is mainly used for clutter removal due to its fast processing. However, different researchers have employed other filters as well. The following Table 1 summarizes different approaches to removing clutter from raw data for vital signs monitoring. 

#### 3.2.3. Vital Signal Detection in Time Domain

The next step is to find the human chest location because the heart and lungs motion can be captured from the signal reflected from the chest area specifically. The individual waveforms reflected from the body are grouped into a matrix of size “m×n”, where variable ‘*m’* stands for slow time length and variable ‘*n’* represents fast time. The slow time sampling frequency of the radar for our experiments is found to be 92 Hz, which is higher than the Nyquist minimum criteria, as the highest frequency of interest in vital signs is 2 or 120 Hz/min. The range of the radar sensor is configurable through the parameter ‘Frame stitches’. After removing clutter and combining the waveforms into a matrix, we have to search for the RR and HR. Fourier Transform is used for conversion from the time domain to frequency domain. However, we do not use the whole matrix for transformation. The column of interest is found in the matrix, which contains the periodic motion caused by the lungs and heart contraction and relaxation cycles. 

**Figure 3:**
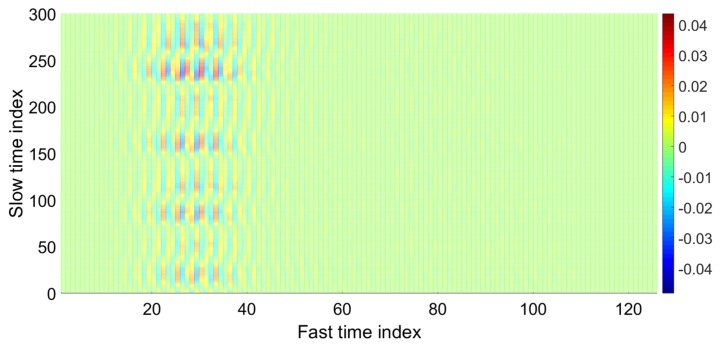
Waveforms after clutter removal.

As shown in Figure 3, the data matrix is represented as slow time vs. fast time with a size of “m×n”. To localize the data received from the chest area in the above matrix, the variance of all columns is calculated, and the column with the maximum value of variance is selected as the fast time distance corresponding to the chest location [11]. The data matrix column with the highest variance (that contains the vital signal) is plotted below. 

**Figure 4:**
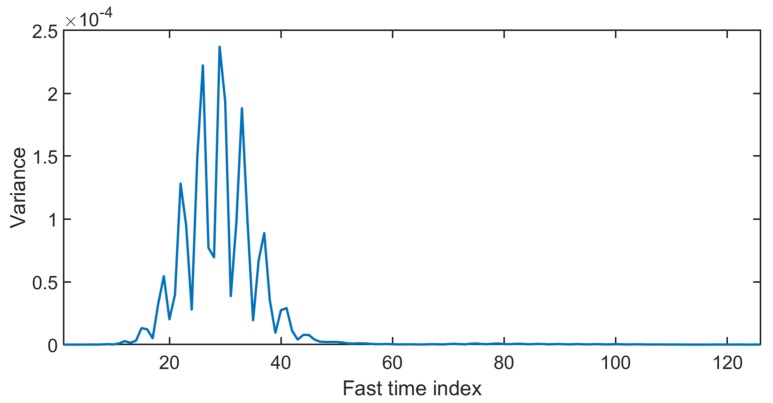
The variance of the signal at different slow time indexes.

**Figure 5:**
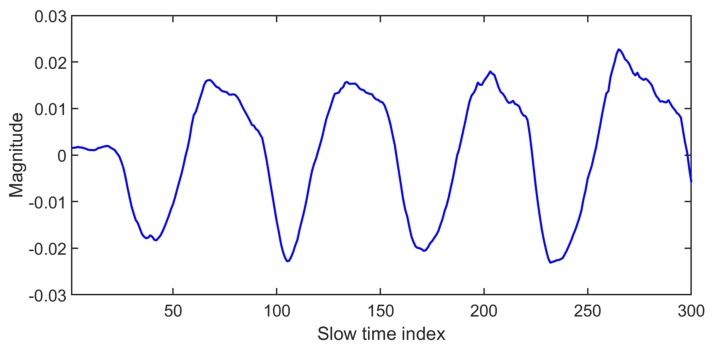
The vital signal in time domain.

In Figure 4, the maximum variance column that represent the fast time location of the human chest is found, and the signal obtained at the location of human chest is plotted in Figure 5. In Figure 5, the signal seems to be cyclic with breathing frequency; however, the smaller peaks at regular intervals show the heartbeat. 

## 4. Respiration and Heart Rate Measurement 

### 4.1. Vital Signs Detection Using Frequency Analysis

The next step is to transform the vital signal to the frequency domain. FFT algorithms are used widely for transformation; however, some researchers also used algorithms such as wavelet transform. 

**Figure 6:**
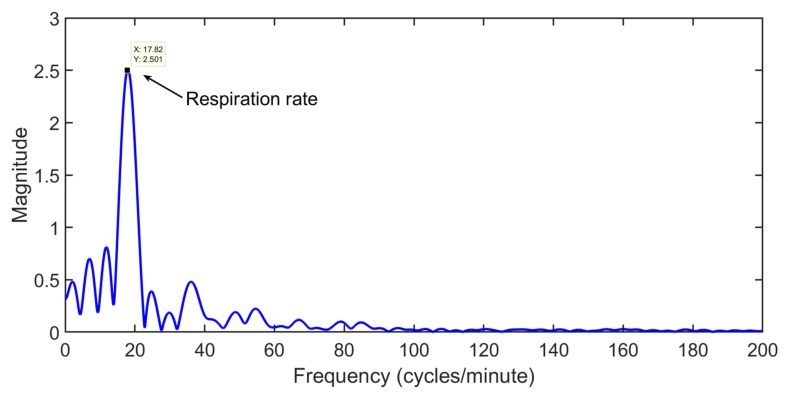
The frequency transformed version of the vital signal.

The spectrum of the vital signal is shown in Figure 6. The strongest peak value is located at a frequency of 0.3 Hz (18 cycles/min). It is the breathing signal because the lungs create the maximum displacement inside human chest. The harmonics of breathing are presented at natural multiples of the breathing frequency, and those harmonics may lie in the region of HR frequencies. In order to suppress the breathing harmonics for accurate heart rate detection, researchers have employed different techniques as follows. In [4,59], the authors employed an MTI filter for breathing harmonics suppression from vital signal spectrum. While the above references use Fourier Transform to find the breathing rate, two other researchers in References [10,62] have used a wavelet transform based algorithm and have shown improved results the in case of breathing measurement using a UWB radar sensor. Contrary to the filter-based solution for heart rate extraction, the authors in [9,60] have used the statistical analysis of the FFT signal to extract the heart rate. Based on the number of occurrences of indexes in the heart frequency range, the heart rate is chosen among the modulation, intermodulation of the breathing frequency and the heart rate. In another study [63], the authors have used the algorithm of a maximum likelihood (ML) based predictor to estimate the vital signs of a person through IR-UWB radar. The results based on proposed algorithm were compared with standard correlation and MUSIC based algorithms. It was shown that the proposed ML based predictor has better performance compared to other existing methods for vital signs measurement. 

In this paper, we mainly discuss signal processing for vital sign detection through IR-UWB radar. However, other two main types of radar sensors used for vital sign detection are Continuous Wave (CW) Doppler [64,65] and Frequency Modulated Continuous Wave (FMCW) radar [66]. The main difference of CW and FMCW radar-based signal processing with IR-UWB radar is the pre-processing of the received signal, as the vital signal information is presented in a different form. In the case of IR-UWB radar, the chest displacement causes a change in the magnitude of the received signal. The data matrix consists of slow-fast time indices. Clutter removal is applied to the range-time matrix, and then spectral analysis is performed as shown in Section 3.2 and Section 4.1. In FMCW and CW radar, the vital signal information is present in the phase of the received signal. For FMCW radar based vital sign detection [64], the received signal is first passed through range-FFT to get the complex range profile. Then the DC components are compensated and phase wrapping is performed to remove static clutters. Another FFT is performed over each column to get a matrix called a range-vibration map. Then the best range is selected based on the average maximum power, followed by spectral analysis to find the RR and HR values. In case of Doppler radar sensing as in Reference [64], the signal is first demodulated, as the chest displacement information due to heart and lungs is present in the phase shift of the received signal. After pre-processing, the vital signs are extracted using the autocorrelation method. 

### 4.2. Previous Work Related to Vital Signs Extraction from Radar Data

Most prominent and early works in the field of algorithms development for vital signs measurement in real time are as follows. In Reference [67], the propagation of a UWB pulse into a layered model of the human body is studied to analyze absorption and reflection of the UWB signal by different tissues of the body. Different time behaviors for the transmitting UWB pulse are considered, and the results are compared with a focus on the feasibility of breathing and heartbeat monitoring. In [4], the feasibility of the vital signs is addressed. The mathematical formulation of the reflected signal containing breathing and heart beat is performed, and the intermodulation of breathing and heart rate signals is also analyzed in detail. Moreover, to detect the heart rate correctly, a filter to suppress the harmonics of breathing signal is proposed. Authors in Reference [57] have presented SVD for the removal of dynamic clutter and an EEMD based frequency accumulation algorithm for breathing frequency. Leib and co-authors have presented an autocorrelation-based receiver to detect vital signs, specifically the heart rate of a human [68]. To improve the resolution of the signal, a Wiener filter was used for deconvolution. Moreover, a wooden board was used for the reflections of the UWB signal, and it was proved that UWB may also be useful for imaging systems because the UWB signal can go inside the wooden board and it is reflected from both the sides of the board. Khan et al. [9] have presented a detailed algorithm for vital signs measurement, including the noise removal using a Kalman Filter (KF) and random body motion detection based on autocorrelation of the vital signal. It was proved that the KF improves the signal to noise ratio (SNR) of the signal, and the motion detection removed some outliers when monitoring was done in real time where the person was free to move his/her hands, body, lips, eyes, etc. Huang and co-authors [69] have shown the effect of different hardware design parameters, such as pulse width and transmitting power, on the vital signs measurements. The study concluded that the SNR of the received vital-sign signals with the pulse width modulated mechanism in high-gain mode are obviously improved. In Reference [61], the authors have addressed the problem of the weak heart signal as compared to the strong breathing harmonics, which makes it difficult to separate the heart signal from the noise and breathing harmonics. They have proposed a method that uses the feature time index with the first valley peak of the energy function of intrinsic mode functions (FVPIEF) calculated by a pseudo bi-dimension ensemble empirical mode decomposition method, which extracts the vital signals by the ensemble empirical mode decomposition (EEMD). In the References [70,71], the authors proposed Harmonic Path and Averaged Harmonic Path algorithms to accurately estimate the vital signs in the presence of breathing harmonics. In another study in Reference [72], a novel noncontract vital sign detection method based on multiple higher order cumulant is presented. According to the characteristic of the vital sign for impulse ultrawideband radar, the quasi-periodic reflected echo in slow-time is analyzed. The novel method is theoretically deduced from fourth-order cumulant. It is proved to be better than the reference fast Fourier transform method by simulation and experiment. In Reference [19], a complementary metal oxide semiconductor (CMOS) based UWB radar is used for vitals monitoring. In order to realize the high rate of sampling, the principle of continuous-time strobed sampling is used as compared to conventional sampling. For each pulse transmitted, the backscattered EM energy is sampled after a given time offset [19]. Richards et al. [73] have proposed a system that alerts when a patient has some abnormal behavior using radio sensors inside a hospital. This system monitors the vital signs as well as the location of the patient inside the hospital building. After locating the patient, a communication between medical personnel and the patient is established. Zito et al. have implemented a system-on-a-chip UWB radar for health monitoring and for safety in emergency situations [74]. An autocorrelation concept was used by Shen et al. [75] for localization of the subject, and a Variational Mode Decomposition (VMD) algorithm is used for measuring the periodic vital signals using IR-UWB radar. Researchers in Reference [76] have used impulse radar for vital signal detection. The multiple automatic gain control (AGC) technique is used to increase SNR, thus enhancing the amplitudes of the breathing signals. The experiments were carried out in different environmental scenarios such as indoor, outdoor and actuator. Averaging filters were employed to increase the SNR value of the respiratory signal. As some of the radar sensors have narrow beamwidth antenna, thus, data might become unavailable if the person in not in the line of sight of the radar. To this end, an important study was conducted by Khan et al. [11] to predict the missing data. They have recovered the missing data obtained through linear prediction techniques during vital signs measurement using IR-UWB radar sensors. 

As many researches have used different algorithms for vital sign monitoring, in order to get a quick overview of these algorithms, we organized the pioneering work in a table with the respective articles and the vital signs extracted in that work. In Table 2, given below, we have summarized the algorithms that are being used for specific vital signs extraction from radar signals. Different parameters such as accuracy, number of subjects involved and type of experimentation are presented in Table 2. 

## 5. Vital Signs of a Non-Stationary Subject

Most of the researchers considered a stationary human subject for their vital signs measurement experiments. However, being idle for long time is unnatural for human beings. Khan et al. [9,78] have used the concept of autocorrelation to find the parts of the nonstationary signal that are contaminated with motion artifacts and removed those parts from the signal to decrease the false detection of heart and respiratory rate measurements. Adjrad et al. [79] have used Empirical Mode Decomposition (EMD) and the Hilbert spectrum (HS) to find the heart and breathing rate of the human. First the signal was decomposed by EMD into Intrinsic Mode Functions (IMFs) to preserve any local property in the time domain, and then the Hilbert transform was used for finding the instantaneous frequency when the subject is nonstationary. Yin et al. [80] have proposed a new algorithm for body movement compensation by IR-UWB radar. The results were verified by Bland–Altman analysis with a mean deviation smaller than 0.1 s. Large body movements were estimated by adopting a distance estimation method using a Kalman Filter and the k-Nearest Neighbor technique. A minimum error rate of 4.6% was achieved as was compared to the standard ECG measurement for the moving state of a human body. In another study [81], the authors analyzed the effect of body movements, such as hand motion and lips movement during speaking. A mathematical model and experimental results are given for stationary as well as non-stationary cases. It is shown that the SNR is degraded in the case of vital signs measurement during speaking and periodic hand waving motions. In a study carried out by Mercuri et al. [82], the authors have implemented an architecture and algorithm for multiple target vital signs measurements using UWB radar sensors. They used a three-stage process to carry out multiple target vital monitoring. Initially, (i) the multiple target tracking algorithm was performed followed by (ii) the removal of the body movement artifacts, and then (iii) the vital signs were extracted. The artifacts were located using the CWT technique and then attenuated by a moving average filter. The respiration and heart rate components were separated using wavelet decomposition. The FFT algorithm was used to extract breathing and heart rates. Lazaro et al. [83] implemented vital signs using a radar sensor. Moreover, the breathing motion and random body motion are classified as micro and macro motion, respectively. A minimum distance threshold was defined to detect static and moving human targets. They showed that since respiration results in a small motion of the human skin so that the human body remains in the same range bin, during random body motion, displacement is larger and the human body position is varied. The motion artifacts part of the signal was removed from the signal followed by vital signs measurement. To lower the effect of the body orientation on measurement, an antenna diversity scheme was proposed. Two receiver antennae were oriented at different angles. The value of the channel with the best SCR was chosen as the final breathing rate. A summary of the above algorithms is given in Table 3 as follows.

## 6. Vital Signs Inside Vehicles

It is very important to monitor vital signs of a driver for safety reasons such as driver drowsiness detection, which can reduce the chances of a car crash by the mistake of a drowsy driver. Yang et al. [61] have done research on measuring the vital signs of multiple targets inside a car. They have used the Variational Mode Decomposition (VMD) method to find vital signs of the driver as well as the passengers using only one radar. Leem and co-authors [30] have shown the feasibility of the vital signs of a driver using IR-UWB radar inside a vehicle. They have also shown that this method can be useful to find the drowsiness of the driver. Yang and co-authors in [84] have studied the in-vehicle vital signs monitoring. They investigated to find the optimum position inside the vehicle using total of 16 positions inside a car. The rear-view mirror was found to be a confident position for vital signs measurement. The authors have implemented the vital signs measurement on an actual on-road car. Haramki et al. [85] have used radar sensors to monitor facial expressions, body parts movements, heart and breathing rate and sweating on the body to continuously observe the driver in order to ensure the safety of the driver and the vehicle. Researchers in Reference [86] have used a penetrating radar for vital signs measurement aimed at vehicular application. Usually the radar waves reflect from the skin, which measures the displacement on the skin surface due to lungs and heart movement, but in this paper, a penetrating radar was used that observes heart displacement on a relatively large scale of millimeters, which makes it easier to monitor the heart rate. They used an aluminum chamber to emulate the automobile environment. The SNR was improved as compared to Doppler and IR-UWB radar with a reflected waves feature. In article [87], researchers have designed a back antenna for vital signs measurement inside a vehicle. As the body reflective coefficient lies around 75%, it is hard to transmit the EM waves through the body tissue. A body coupled antenna was designed to reduce reflections and improve body penetration of radar EM waves. Performance comparison of the above algorithms related to in-vehicle vital sign detection is presented in Table 4.

## 7. Sleep Monitoring 

Almost one third of the population suffers from difficulty while falling asleep, frequent waking during sleep, poor sleep quality and many sleep-related breathing problems [88,89]. For aged people, there are concerns that medical emergencies during sleep might go unnoticed. Unfortunately, most current devices for sleep monitoring are uncomfortable and are used primarily for making medical diagnoses. However, many health benefits could result from unobtrusive sleep monitoring in a home environment. Many researchers have used IR-UWB radar for non-contact sleep monitoring. Ziganshin and co-authors [90] have done a study on sleep apnea detection of babies with UWB radar. They have proposed a device called “NanoPulse Baby SleepGuard”, which monitors the health using radar and is also equipped with a temperature sensor and microphone. The radar mainly monitors body motion, breathing and heart rate of the baby. It gives alarm when a dangerous level of gap in breathing is detected and, hence, prevents the occurrence of Sudden Infant Death Syndrome (SIDS) [90]. Tataraidze et al. [91] has studied vital monitoring during sleep. They have performed a lot of experiments to differentiate between distinguish REM and non-REM sleep only by breathing pattern recorded by bio-radar without applying any additional contact sensors. A robust method for overnight monitoring of vital signs using low power radio waves is presented by Li et al. [92]. In addition to theoretical analysis, the authors have given measured data as a proof that the monitoring from the back of a body is advantageous [92]. Researchers in a recent study in the Reference [93] have shown that by using UWB radar and CNN, different sleep situations such as Eupnea, Bradypnea, Tachypnea, Apnea and Motion can be classified from the signal data. Javaid et al. [94] worked on detecting sleep apnea using an under-mattress IR-UWB radar and machine learning signal processing. Normal and apnea epochs were extracted from the IR-UWB data. Using these epochs, statistical features were derived and a Linear Discriminant classifier was trained. The accuracy of the system was around 70% for apnea detection. In a study related to sleep monitoring using IR-UWB radar [95], authors have measured the total body movement as well as respiration during sleep from the radar signals by forming range-frequency-power matrices. The matrices are generated using the FFT algorithm on data from each 5 cm distance increment. A 3 s window is used for the body movement detection, while a 20 s window is used for detection of the respiration rate. The sleep algorithm then summarizes the movement index and respiration rate values into 30 s epochs. The threshold values are applied to find the sleep and wake status of a person. The mean accuracy for discrepancy between the radar and PSG was 0.931, which is better when compared to reported actigraphic recordings. Hung et al. [96] used UWB radar for a variety of monitoring activities such as micro movement, vitals measurement and sleep apnea detection. The sensor was attached to a smart mattress for long term physiological monitoring. In this study, an adaptive and digitalized post calibration technique was used to increase the sensing accuracy and achieved the micro movement detection. 

## 8. Through the Wall Vital Signs

Many researchers have used the respiration detection capability of radar as a human presence detection behind a wall. A study has used pulse radar and continuous transform for signal processing to find a human presence behind the wall by detecting their respiration [97]. Another research used the surface penetrating radar to record the pulse beating and breath motion to find a human behind an obstacle [98]. Levitas et al. have shown that the human body can be detected and localized using the breathing and weak heart rate pulses through UWB radar sensor behind the wall. They have used a radar with large operational bandwidth (11.7 GHz) [99]. Yan et al. have used an IR-UWB radar to find the respiration of a human at a distance of 0.7–2.5 m behind a concrete wall for human detection [12]. The RADAR Flashlight was designed to detect the respiration of a human subject behind a wall, door or an enclosed space with non-conductive walls [100]. In Reference [101], the authors have designed a UWB linear array to obtain life information in the rubble cases. This method also estimates the azimuth information of a human subject along with the respiratory motion estimation. It can also extract vital signs of multiple persons behind the wall. In another study, Yan et al. [102] have implemented the Variational Mode Decomposition (VMD) algorithm for through-wall target vital signs tracking using an IR-UWB radar sensor. In the experimental section, a 0.15 m thick wall was used for evaluation of the through-wall respiration detection proposed in the paper. To show the accuracy of the algorithm, three human subjects were placed at the same distance and three different respiration patterns, i.e., constant, piece-wise constant and time varying breathing rate, were measured. A quick overview of the above algorithms is given in Table 5.

## 9. Vital Signs of Neonates 

Contactless vital signs monitoring is very useful for babies because it is very hard to use the cumbersome ECG wires around the body of a small baby. Impulse radar has been used by many researchers to monitor the breathing and heart rate. A CMOS UWB pulse radar was developed for monitoring the vital signs of an adult as well as a baby [103]. In Reference [104], the authors have performed vital signs monitoring for neonates in order to check the feasibility. The authors have compared the accuracy and reliability of radar measurements with those of conventional impedance pneumography measurements [104]. One such research work carried out by Mahbub et al. [105] has designed an IR-UWB transceiver to be used for the remote monitoring of respiration signals and apnea detection in a non-invasive way for premature infants. The transmitter is designed and fabricated in a 130 nm standard CMOS process that consumes extremely low power of 9.12 µW. Huang et al. [106] have used UWB radar to detect apnea of an infant based on the respiration signal. If the respiration signal is absent for a certain time, then the apnea detection warning is issued. Moreover, to overcome the movement problem of the body of infant, a localization algorithm is implemented to constantly check the location of the infant before measuring the respiratory signal. In Reference [107], a correlation-based hardware demonstrator was presented. At the receiver end, the cross-correlation concept was applied and custom-built ICs were used in the transceiver along with already available commercial components on an antenna substrate. Respiration rate was measured for two male humans and an infant to show the accuracy and safety of the proposed system. Performance of the above algorithms related to neonate vital signs assessment is presented in Table 6.

## 10. Other Medical Applications 

In this section, a brief introduction of the usage of UWB in medical imaging and fall detection is given as follows.

### 10.1. Medical Imaging

Research on the biomedical applications of UWB radar refer to the design, development and clinical testing of UWB technology for different medical applications [109]. Applications of UWB devices include: cardiac biomechanics assessment, chest movements assessment monitors for breathing, soft-tissue biomechanics research, heart imaging and chest imaging. UWB is also used for medical imaging to diagnose different conditions such as tumors. 

Most research work of microwave imaging deals with early-stage breast tumor detection. While microwave radar does not offer high spatial resolution like X-rays, it exploits some physiological parameters of clinical interest such as water content, vascularization, blood flow and temperature, as it can identify and localize dielectric contrast [22]. UWB based imaging has advantages such as low cost and less power requirements; however, it comes with its own challenges. Firstly, since the composition of permittivity and conductivity are inhomogeneous, it may result in ambiguous conclusions. Secondly, geometric properties, such the internal structure of tissue, may be irregular on grossly differing length scales [37]. The ongoing research is motivated by the need for early stage tumor detection through UWB with high specificity and sensitivity [36]. Microwave imaging can detect tumors as small as 1–2 mm [110]. Hagness et al. [22] have developed microwave miniature radars to detect breast malignant tumors. Researchers in Reference [21] used confrontal microwave imaging for the detection and localization of tumors using a radar sensors array. Lazaro et al. have used UWB microwave imaging for the detection of breast tumors using wavelet transformation [111]. A method is proposed for locating tumors that is based on time-of-flight of the signal backscattered by the tumor. Time-of-flight is detected using a wavelet transform algorithm. Researchers in Reference [112] presented a computationally efficient image reconstruction algorithm to detect breast cancer of a human. In the experiments, a sample of tumor that has a 6 mm diameter and a depth of 3.3 cm was used. The signal to clutter ratio was defined as the ratio of the tumor response peak to the maximum clutter response in the breast interior, and that value was calculated to be approximately 8 dB for the breast model with a tumor of 2 mm in diameter. In another study, Kikkawa et al. [113] have developed an IR-UWB CMOS circuit for breast cancer detection. A Gaussian mono pulse generator and transmitter antenna were used at the transmitter side. A three-stage cascade topology based Low Noise Amplifier (LNA) was used at the receiver side to improve the SNR of the signal with a voltage gain of up to 23 dB at 7.5 GHz and 3 dB bandwidth. Confocal imaging was carried out by using the CMOS chipset. 

Another study by McEwan has used pulse radar for detecting, monitoring and measuring the movement of the heart, lungs, other body organs, tissues and members, and for processing the corresponding bio-potential signals [114]. An approach for the benefit of high and ultra-high field magnetic resonance imaging (MRI) and other applications, e.g., intensive care medicine and biomedical research using UWB signals, was presented in Reference [115]. Saha et al. [116] have used CMOS IR-UWB radar for application to medical imaging. They have employed a UWB transmitter that has a flexible pulse rate with extremely low power. The circuit was designed with 130 nm CMOS RF technology. The transmitter can generate pulses at a low speed and, hence, it can be used for imaging in healthcare. However, a high speed pulse may be useful for short range communication. 

### 10.2. Fall Detection 

These days, many societies suffer from problems within the aging population. Medical costs of such populations are increasing due to the hospitalization of patients. A sudden fall in a room or bathroom is one of the causes of hospitalization of senior citizens. If senior citizens are continuously monitored in their homes using non-invasive sensing technology such as radar sensor, then their life quality can be considerably improved by detecting an illness in its early stages and detecting emergency situations such as fall or irregular breathing or heartbeat. In Reference [117], the authors have used an IR-UWB transceiver to find a range and motion estimation. Using the range and motion estimation, the different states of a person can be detected, such as “sleeping in bed”, “sitting up in bed”, “falling down”, “wandering in room” and “going out and inside the room”. The fall detection rate was found to be 95% [117]. Researchers in [118] have achieved good results in fall detection by using a micro-motion signature and unsupervised learning, with sensitivity and specificity greater than 97% and 90%, respectively. Other researchers [119] have used range information integrated with a fall detection algorithm to distinguish an actual fall from a sitting motion in order to reduce the false alarm rate. It concluded that an actual fall exhibits twice the range extent as that of sitting. Mercuri et al. [120] presented a complete system that combines wireless communication and data processing techniques for remote health monitoring. They have demonstrated an adequate detection of the target’s absolute distance and a success rate of 94.3% in distinguishing fall events from normal movements. 

## 11. Conclusions 

In this paper, literature related to medical applications of IR-UWB radar, mainly vital sign monitoring of a human, is discussed. Different scenarios are discussed, such as vital signs monitoring of a stationary human, sleep monitoring and driver vitals monitoring for safety purposes. In each section, we have also mentioned in detail the signal and image processing algorithms used for the targeted applications. Recent research trends in vital signs assessment through radar are discussed, and it is concluded that most researchers are now interested in working on the robustness of algorithms that are used for vital sign extraction. The main challenge that researchers face and that limits the wide use of this technology in hospitals is the effect of motion on algorithms performance. Researchers are giving different solutions for the assessment of vital signs of a non-stationary human subject. If this problem is overcome, then this heath monitoring technology will not only be widely used in hospitals, but also in cars while moving on road. Another factor that may be considered for future research is the heart rate measurement of obese people, as it is known that chest displacement due to heart beat may be negligible in obese people. Medical imaging and fall detection through radar are also very hot research topics, and it would be useful because of the growing number of aged people all over the world. To sum up, UWB radar technology is one of the few technologies that may gain more and more popularity in the digital health industry.

## Figures and Tables

**Table 1:** Clutter removal algorithms for vital sign detection.

Clutter & Noise Removal Algorithms	Research Articles
Loop Back Filter	[7,9,10,25,27,28,29,30,38,54,55,56]
Singular Value Decomposition (SVD)	[57]
Kalman Filter	[58]
Single Delay MTI Filter	[42]
Averaging Method	[4,17,18,52,59,60]
Pseudo-Bi-Dimensional Ensemble Empirical Mode Decomposition (PBDEEMD)	[61]

**Table 2:** Performance comparison of vital sign detection algorithms.

Vital Sign Assessed	Vital Sign Algorithms	Research Articles	Experimental Setup Range/Subjects/Reference Measurement Method	Results
**HR, RR**	Fast Fourier transform (FFT)	[47,52,60]	[47] 0.5–1 m/16/ECG [60]/9 human /fingertip pulse oximeter [52] 15 feet/7 humans/electronic heart-monitor	[47] Error rate: 5% [60] 1%–5% [52] Mean error for obstructed RR: 0.3 bpm Mean error for obstructed RR: 0.14 bpm Mean error for unobstructed HR: 8 bpm
**HR, RR**	Ensemble empirical mode decomposition (EEMD) and Continuous wavelet transforms (CWT)	[12]	0.2–5m/3 human/ECG	SNR improvement for RR: 7.5 dB SNR improvement for HR: 4.8 dB
**RR**	Wavelet transform	[8,62]	[8] 6 –14 m/2 males, 2 females, 1 actuator/FFT method measurements [62] 1–2.5 m/-/manual measurement	[8] SNR: (−4.91dB–(−8.28 dB) Deviation: 0.66%–0.24% [62] Deviation: 0.19 per minute
**HR, RR**	MTI and Chirp Z-transform (CZT)	[4]	1 m/3 human/ECG	Error rate BR/HR: 1%–2.4%
**RR**	Multiple Higher Order Cumulant (MHOC)	[72]	2–7 m/1 human/manual	SNR improvement: 13.8 dB
**HR, RR**	HAPA (harmonic path)	[70]	5–15 cm/1 male/pulse-oximeter	Error rate (MSE) for HR: 1.83%
**HR, RR**	Spectrum-Averaged Harmonic Path (SHAPA)	[71]	5–15 cm/8 human/pulse-oximeter	Error rate: 16% improvement over [70]
**RR**	IIR filter	[18]	1 m/--/respiration monitor belt (RPM)	Correlation co-efficient: 0.909 Absolute error: 0.5 mHz
**HR**	Time Domain Processing Algorithm	[16]	0.25–1.25 m/5 human/ECG	Error rate: 1.01%–4.32%
**HR**	Time series analysis	[15]	1m/apparatus/--	Error rate: 1.26%
**HR, RR**	Pulse-Doppler signal processing technique	[19]	5–15 cm/13 human/Polysomnography (PSG)	Deviation: 5%
**HR**	Maximum likelihood period estimation	[63]	0.3 m/1 human/wearable sensor	Mean Square Error: < −8dB
**RR, HR**	Ensemble empirical mode decomposition (EEMD)	[57,61]	[57] 3 m–16 m/5 humans/-- [61] 50 cm/--/human tissue model	[57] Error rate: 1.5–3.75% [61] Error rate: 1.12%
**RR, HR**	Harmonic Multiple Loop Detection (HMLD)	[77]	0.64 m/5 male, 5 female/pulse oximeter (HR), manual chest wall count (RR)	Error rate (RR): 4.95% Error rate (HR): 5.06%

**Table 3:** Performance comparison of vital sign detection algorithms for non-stationary subjects.

Vital Sign Assessed	Vital Sign Algorithms	Research Articles	Experimental Setup Range/Subjects/Reference Measurement Method	Results
**HR, RR**	Fast Fourier transform (FFT), Autocorrelation	[58]	1–2 m/5 humans /ECG	RMSE for RR: 0.006 HR: 0.372
**HR, RR**	Wavelet, Kalman filter	[80]	1–4.5 m/4 humans/ECG	Error rate: 2.25%–4.6%
**RR**	FFT	[81]	70 cm/1 humans/manual	SNR (stationary): 20 dB SNR (speaking): 15 dB SNR (handwriting): 16 dB
**RR, HR**	FFT, Continuous Wavelet Transform (CWT)	[82]	5.4 m/8 humans/ECG	Success rate: RR: 94% HR: 89%
**RR**	FFT, distance deviation threshold (for random body motion detection)	[83]	1 m/22-year-old male/microphone	Normal breathing, apnea, macro-motion detection

**Table 4:** Performance comparison of vital sign detection algorithms for in-vehicle monitoring.

Vital Sign Assessed	Vital Sign Algorithms	Research Articles	Experimental setup Range/Subjects/Reference Measurement Method	Results
**HR, RR**	Location based Variational Mode Decomposition (VMD)	[61]	Up to 1.5 m (inside car) /2 human/Oximeter	Error rate for only driver HR: 7.34% Error rate for two targets in car HR: 6.9%–11.5%
**RR**	FFT, band pass filter	[84]	Inside car/ 3 male, 1 female/manual push button based method	Mean error: 1.06 breathing rate per minute
**RR, HR**	FFT, Data fitting method	[16]	Inside car/5 humans/ECG, respiration belt	Mean RR error: 0.2–0.7 beats per minute Mean HR error: 0.6–2.5 beats per minute
**RR, HR**	FFT, band pass filter	[86]	Inside chamber/ 1 human/ECG	SNR: 8.6 dB
**HR**	FFT, FIR Kaiser filter of order 600, Correlation method	[87]	Inside car/3 humans/3 lead Olimex EKG (for ECG), a BioHarness 3 (RR)	Phase method: 13.1%–22.5% Correlation method: 14.4%–30.3% Slow time method: 24.3%–39.4%

**Table 5:** Performance comparison of vital sign detection algorithms for through-wall monitoring.

Vital Sign Assessed	Vital Sign Algorithms	Research Articles	Experimental Setup Range/Subjects/ Wall Thickness	Results
**HR, RR**	CWT, background subtraction method	[97]	1–5 m (non-obstructive), 0.8 through the wall/50 cm	SNR: 14 dB
**Detection**	Raw data, Frequency spectrum	[98]	1 m/1 human/10 cm	Person detection: 100%
**Detection**	Time domain (slow time signal) analysis	[99]	2.6 m/1 human/wall	Respiratory pattern
**RR**	Spectrum analysis	[12]	0.7–2.5 m/1 human/20 cm reinforced concrete wall	Error rate: 0.6%
**RR**	IIR band pass, moving averaging filter, advanced normalization method, FFT	[101]	5.5 m/3 humans/…	Detection of RR
**RR**	Variational mode decomposition (VMD)	[102]	1.5 m/3 humans/15 cm thick concrete wall	Correlation: 97.6%

**Table 6:** Performance comparison of vital sign detection algorithms for neonates monitoring.

Vital Sign Assessed	Vital Sign Algorithms	Research Articles	Experimental Setup Range/Subjects (Age)	Results
**RR**	FFT	[103]	30–45 cm/3 humans (1 man, 1 woman, 1 baby (5 months old)	Sub-centimeter chest moment detected successfully
**RR**	FFT	[104]	35 cm/9 babies (age 2–27 days)	Mean bias: 1.7 bpm
**RR, HR**	Peak detection method	[108]	60 cm/babies (1–3 years old)	Apnea detected
**RR**	FFT, band pass filter	[106]	1 m/1 infant	1 RR detected at 0.62 Hz
**RR**	Fourier Transform analysis	[107]	20–25 cm/male human, 1 infant (7 weeks old)	Continuous breathing and arrhythmic breathing classification
